# Melatonin in Clinical Practice: Grey Zones Between Chronobiology, Insomnia and Consumer Supplementation

**DOI:** 10.3390/clockssleep8030038

**Published:** 2026-06-24

**Authors:** Alexandros Kalkanis, Aliki Karkala, Athanasia Pataka

**Affiliations:** 1 Department of Pulmonology, Leuven University Center for Sleep and Wake Disorders (LUCS), University Hospitals Leuven Campus Gasthuisberg, Herestraat 49, 3000 Leuven, Belgium; alexandros.kalkanis@uzleuven.be; 2 Laboratory of Respiratory Disease and Thoracic Surgery (BREATHE), KU Leuven University, Herestraat 49, 3000 Leuven, Belgium; aliki.karkala@uzleuven.be; 3 Respiratory Failure Unit, General Hospital of Thessaloniki “G. Papanikolaou”, Aristotle University of Thessaloniki, Exochi, 57010 Thessaloniki, Greece

**Keywords:** melatonin, insomnia, circadian rhythm sleep–wake disorders

## Abstract

Melatonin occupies a paradoxical position in contemporary sleep medicine: despite its physiological role as a regulator of circadian timing, it is frequently used and perceived as a nonspecific “natural” hypnotic. Although melatonin demonstrates modest benefits for sleep initiation and clearer efficacy in circadian rhythm sleep–wake disorders, its clinical use is often undermined by diagnostic imprecision, inappropriate dosing, mistimed administration, inconsistent formulations, and inadequate patient counseling. Circadian disorders can be misclassified as primary insomnia, leading to symptomatic treatment approaches that fail to address the underlying phase misalignment. At the same time, supraphysiological doses and reflexive bedtime administration have become normalized despite evidence that melatonin acts primarily as a chronobiotic whose effects depend more on timing than dose. Regulatory inconsistencies and substantial variability in over-the-counter preparations further complicate safe and reproducible use. These factors contribute to avoidable treatment failure, inaccurate labeling of nonresponse, and persistent misconceptions regarding melatonin’s mechanism of action. Therefore, melatonin should be approached as a pharmacological intervention requiring the same diagnostic rigor, individualized dosing, and longitudinal assessment expected of other sleep therapeutics, particularly when integrated with behavioral and circadian interventions.

## 1. Introduction

Melatonin (N-acetyl-5-methoxytryptamine) is a neuroendocrine hormone synthesized primarily by the pineal gland and secreted into the circulation according to a circadian rhythm regulated by the suprachiasmatic nucleus of the hypothalamus. Its production peaks during the biological night and is suppressed by light exposure, making it a key signal of circadian timing and an important regulator of circadian rhythms. Since its identification, it has occupied a unique position at the interface of basic chronobiology and clinical sleep medicine [1]. The growing scientific and commercial interest in melatonin [2] reflects the increasing recognition of circadian biology as a determinant of health and disease.

The translation of melatonin from a physiological marker of circadian timing to a broadly marketed sleep aid was strongly influenced by claims that age-related insomnia results from declining endogenous melatonin production. Popularized in the 1990s through publications such as “The Melatonin Miracle”, this narrative suggested that melatonin supplementation could function as a form of hormone replacement therapy capable of restoring youthful sleep patterns. However, the scientific basis for this hypothesis remains weak. Although endogenous melatonin secretion declines during early adulthood, evidence indicates that levels remain relatively stable throughout middle and older age rather than continuing to decrease progressively [3]. Furthermore, clinical studies have failed to demonstrate that insomnia in older adults is primarily attributable to inadequate melatonin production [4]. Consequently, the widespread assumption that age-related sleep disturbances can be explained by melatonin deficiency represents an oversimplification of a complex and multifactorial condition.

In sleep medicine, however, melatonin illustrates a persistent disconnect between biological evidence and clinical use. Originally conceptualized as a hormonal signal of circadian phase rather than a sedative, its clinical application has gradually shifted toward that of a mild “natural” hypnotic, a perception reinforced by over-the-counter availability, a favorable safety profile, and widespread commercial positioning [2]. As a result, melatonin occupies an unusual position: a hormone that is often prescribed and self-administered with the informality of a nutritional supplement rather than the caution typically associated with pharmacological agents.

## 2. Evidence Based Indications

Exogenous melatonin occupies an ambiguous role in insomnia management, positioned between a chronobiological intervention and a symptomatic hypnotic, as patients often expect rapid sedation and clinicians in all levels of healthcare frequently recommend it casually as a general sleep aid [5,6]. Although evidence suggests overall benefits across diverse populations and sleep outcomes [5], its effect on total sleep time remains inconsistent [7]. Interpretation of the current literature is further limited by methodological weaknesses and substantial variability in dosing and formulations, preventing meaningful dose–response conclusions [5]. In acute insomnia, evidence is additionally constrained by the practical difficulty of participants’ recruitment and intervention within a narrow therapeutic window [8]. These uncertainties are reflected in the variation across countries between official guideline recommendations and physicians’ real-world clinical practice, shaped by individual judgment. The American Academy of Sleep Medicine does not recommend routine melatonin use for sleep-onset or maintenance insomnia and provides no detailed dosing guidance [9], despite melatonin being a common self-treatment option [6]. In Europe, most countries follow the European Sleep Research Society guidelines for adults, which support prolonged-release melatonin for patients over 55 years old (2 mg, 1–2 h before bedtime) [10]. However, some national scientific societies already consider melatonin a first-line option when a hypnotic is indicated in this population [11,12]. Discrepancies also persist between official guidelines and prescribing rules among European health authorities, particularly regarding long-term use [10] and insomnia comorbid with other conditions [13].

In circadian rhythm sleep–wake disorders, melatonin has a clearer mechanistic rationale and more consistent therapeutic signal than in primary insomnia, as in the latter the benefits are smaller and less predictable [14,15]. Melatonin acts primarily as a chronobiotic rather than a hypnotic: when timed relative to endogenous dim-light melatonin onset, it can advance circadian phase, particularly in delayed sleep–wake phase disorder (DSWPD) [16]. Current guidelines support strategically timed melatonin for DSWPD, emphasizing the importance of timing over the dose, but providing no single standardized-dose regimen [17]. A major clinical pitfall, however, is the superficial management of circadian disorders, in which melatonin is prescribed as a stand-alone sleep aid. Without assessment of circadian phase, light exposure, and behavioral contributors, melatonin is often used as a nonspecific sleep aid rather than as a circadian intervention. Its efficacy depends largely on administration at the appropriate circadian time, which varies according to the therapeutic goal (e.g., phase advance, phase delay, or circadian entrainment), and on coordination with behavioral and light-based measures [2,18]. Such measures usually includes keeping a consistent sleep–wake schedule, building a wind-down routine, exercising regularly, and avoiding late caffeine, alcohol, heavy meals, and bright light or screens [19,20]. Otherwise, treatment may mask rather than correct the underlying disorder. Similar limitations apply when melatonin is used without structured protocols coordinating sleep timing, light, and darkness, in other circadian misalignment disorders [21]. In shift work disorder, it has been proposed as part of a broader strategy to compensate for insufficient evening darkness [22]. In jet lag, it may facilitate circadian realignment, particularly after eastward travel across five or more time zones [23,24], when used in short-term, protocolized regimens [25]. However, much of the evidence is dated, and uncertainty regarding optimal dosing, formulation, and timing limits generalizability.

Finally, melatonin supplementation has also shown promise in idiopathic REM sleep behavior disorder when administered at a fixed, chronotype-adjusted clock time [25]. Beyond sleep medicine, melatonin has also been investigated across multiple inflammatory, neurological, and oncological contexts; however, these applications remain outside the scope of routine sleep-focused prescribing and should not be used to justify casual supplementation [26,27,28,29,30].

## 3. Overlap in Clinical Presentation

In routine practice, circadian disorders can be mistaken for sleep-onset insomnia, particularly in adolescents and young adults, leading to treatments that do not target the underlying mechanism [31,32,33]. While both these conditions present with difficulty initiating sleep at socially conventional times, DSWPD reflects a delay in endogenous circadian phase, whereas primary insomnia is typically characterized by hyperarousal and impaired sleep regulation [2]. Early clinical descriptions already underscored this distinction, showing that patients labeled with sleep-onset insomnia often exhibit normal sleep duration and quality when allowed to follow their preferred delayed schedule [34], highlighting that the complaint may arise from misalignment rather than intrinsic sleep dysfunction. In contemporary cohorts, this distinction remains blurred. Objective assessments reveal that classical markers of insomnia, such as prolonged sleep latency on polysomnography, are not consistently present in individuals diagnosed with DSWPD based on sleep diaries and actigraphy, suggesting that behavioral and psychological factors frequently coexist and complicate phenotyping [33]. However, reliance on subjective reports alone is also insufficient, as the disorders can intersect or evolve over time [35]. Diagnostic categories may therefore oversimplify presentations that arise from multiple interacting mechanisms. For example, difficulty initiating sleep may reflect circadian misalignment, conditioned hyperarousal, or a combination of both, underscoring the need for careful phenotyping when selecting treatment strategies.

Parasomnias may similarly be misperceived or reported as sleep-maintenance insomnia, as repeated awakenings can fragment sleep time [25]. Moreover, current adult data show that parasomnias and insomnia symptoms frequently coexist, as both often arise in the context of mood disorders [36], other sleep conditions and as a result of medication effects [37]. Insomnia can also act as a trigger for parasomnias, further complicating differential diagnosis [38], mainly by fragmenting sleep and increasing arousals that precipitate episodes [36]. Sleep deprivation arising from insomnia, could also increase complex behaviors occurring in slow-wave sleep [39]. Clinically, a careful history is essential to distinguish insomnia from parasomnias and other sleep or psychiatric disorders, while video-polysomnography is reserved for cases in which another sleep disorder is suspected [40]. In the absence of longitudinal sleep–wake assessment and, when feasible, objective circadian phase markers such as dim-light melatonin onset (DLMO), misclassification of the underlying disorder may occur, resulting in inappropriate hypnotic use, mistimed melatonin administration, and avoidable treatment failure. Nevertheless, the clinical application of melatonin remains challenging, as findings regarding the relationship between circadian phase markers and sleep timing are inconsistent. While several studies have reported significant associations between DLMO and sleep parameters [41], others, including recent data from healthy adults, have failed to confirm robust associations between DLMO and sleep onset or sleep offset [42], suggesting that circadian phase assessment should be interpreted alongside longitudinal sleep–wake patterns and clinical context.

## 4. Dose-Mechanism Mismatch in Melatonin Therapy

A common but often overlooked cause of inappropriate melatonin use in clinical practice is dose escalation beyond physiologically justified ranges. In both Europe and North America, doses of 3–10 mg have become normalized, despite evidence that such amounts produce supraphysiological plasma concentrations for most chronobiological indications [2,43]. Experimental data indicate that doses between 1 and 5 mg can elevate circulating melatonin levels 10- to 100-fold above endogenous nocturnal concentrations, whereas doses of approximately 0.1–0.3 mg more closely replicate physiological conditions [44]. The therapeutic rationale for conservative dosing is not sedation, but circadian phase-shifting, a mechanism that appears to operate optimally at near-physiological concentrations. Low doses (0.3–0.5 mg) improve sleep efficiency while maintaining physiological melatonin dynamics [45,46]. In contrast, 3 mg results in prolonged daytime melatonin elevation and altered sleep architecture despite perceived sleep improvement [47,48]. Specifically, prolonged-release 3 mg at bedtime has been shown to sustain high melatonin levels for up to 12 h [48].

Collectively, these findings raise the question of whether higher doses provide superior therapeutic outcomes and suggest that current prescribing and consumer practices may reflect market-driven trends rather than evidence-based pharmacology. Although melatonin is generally considered well tolerated, higher doses may increase the likelihood of adverse effects without proportional clinical benefit [39]. Furthermore, the relative scarcity of long-term randomized placebo-controlled trials warrants caution, particularly in potentially vulnerable populations such as children, older adults, and pregnant women [49,50,51,52,53].

Another often overlooked dimension is the fragmented regulatory framework governing melatonin across Europe. In the European Union, products containing ≥2 mg are regulated as medicinal products under European Medicines Agency oversight, whereas lower-dose preparations are commonly marketed as food supplements with less stringent requirements for standardization and clinical supervision [54,55]. This dose-based distinction creates a regulatory gray zone in which consumers can obtain melatonin over the counter [56], online, or through cross-border transport [57] without adequate guidance on indication, formulation, timing, or cumulative exposure [56,57]. Formulation variability adds further uncertainty. Preparations differ in release profile, route of administration, pharmacokinetics and product quality, resulting in inconsistent therapeutic effects and unpredictable plasma concentrations [58]. Oral melatonin has low and highly variable bioavailability, influenced by age, smoking, caffeine intake, comorbidities and concomitant medications [59,60]). Immediate-release, prolonged-release, sublingual and buccal formulations also differ substantially in absorption and duration of action, with some prolonged-release products maintaining elevated melatonin levels for extended periods of time [61]. Most importantly, analyses of commercial supplements have found melatonin contents ranging from 83% to 478% of labeled doses, raising concerns about quality control and reproducibility [58], and further blurring the distinction between supplements and medicinal products under Europe’s dose-based regulatory framework [55,58]. Although melatonin generally has a favorable safety profile, these inconsistencies undermine its reliability and challenge the widespread perception that all formulations are clinically interchangeable [62,63].

A further source of pharmacological misuse is the often-inappropriate timing of administration. As noted above, melatonin is not a conventional hypnotic but a chronobiotic, with effects that depend on the timing of administration relative to the individual’s circadian phase. When low dose (<2 mg) immediate release melatonin is taken consistently in the early evening, several hours before habitual sleep onset, it can advance circadian timing and facilitate earlier sleep in both healthy individuals and DSWPD patients, as shown in experimental and clinical studies [46,64]. The optimal timing of administration may, however, differ according to the specific circadian rhythm sleep–wake disorder being treated [64]. By contrast, the common practice of taking melatonin “at bedtime” or intermittently on an as-needed basis, reflects a sedative model of use that may reduce efficacy or even produce the opposite effect, contributing to the perception that melatonin worsens baseline sleep [2]. Formulation-specific pharmacokinetics further complicate use: immediate-release products act within approximately 45 min, whereas prolonged-release preparations reach peak concentrations later and may maintain elevated plasma levels for up to 8 h [44,65]. When poorly timed, these profiles may extend melatonin exposure into the biological daytime and alter sleep architecture, particularly at higher doses [47]. Although community pharmacists are well placed to counsel on timing, regularity of administration, and especially advise against combination with other medications [17,66], dispensing patterns of 3–6 mg daily [66] suggest that current practice may reinforce non-chronobiological use. More importantly, studies have shown that although pharmacists are among the most accessible healthcare professionals and frequently provide guidance on melatonin use, many lack detailed knowledge of its pharmacology and appropriate patient selection [66,67].

This is further complicated by the fact that, in practice, there is no specific advice given to the patients from their provider regarding the titration process. Most studies use fixed doses, but several reviews and trials give practical ranges and how to adjust them. However, they differ by age, formulation, or indication [55,68,69]. Pediatric and national guidelines, including United Kingdom’s and Dutch recommendations, propose stepwise titration schemes tailored to circadian goals rather than conventional hypnotic use [70,71]. According to them, a brief, structured trial is usually sufficient and when timing is correct, clinically meaningful effects typically emerge within one to two weeks [68]. Dose–response analyses indicate that benefits plateau at relatively modest doses (approximately 2–4 mg in children and around 4 mg in adults) while higher doses offer little additional benefit [72,73]. Furthermore, while guidelines endorse use up to 13 weeks [9,10], they focus on its effects and on withdrawal from hypnotics [74], not on structured tapering regimens for melatonin itself. Across adult and pediatric studies, prolonged-release melatonin, even when used long-term, has not been associated with physiological withdrawal or significant symptom rebound [75,76]. Nevertheless, because melatonin is often prescribed with minimal guidance owing to its favorable safety profile, these should be interpreted as context-specific and not as justification for indiscriminate dose escalation. Overall, the available evidence suggests that optimizing timing and using the lowest effective dose is more consistent with melatonin’s chronobiological mechanism than adopting a conventional titration approach [2,62].

## 5. Issues with Compliance

All of these grey zones and uncertainties in the prescription and administration of melatonin could be eventually predisposing the individual for treatment failure. Because melatonin is frequently prescribed or self-initiated with minimal guidance [45], patients may receive insufficient instruction on dose, timing, consistency, and the duration required for an adequate trial. Moreover, melatonin follows first-order kinetics, meaning that higher doses produce proportionally higher plasma concentrations, which increases interindividual variability and reduces dose–response predictability [77]. As melatonin is often used as an adjunct or alternative in patients who cannot tolerate conventional hypnotics [10], its chronobiotic properties may be mistaken for sedative effects. In many cases, the apparent lack of efficacy reflects regimen error rather than true pharmacological non-response [46,66,68]. When these expectations are not met within a few nights, treatment might be discontinued before sufficient time has been allowed for the medication to take effect. If such sub-optimally implemented regimens are subsequently recorded as “ineffective”, the medical record may acquire a permanent but misleading label of treatment failure, discouraging future use and excluding a potentially useful therapy. In this sense, melatonin is often judged ineffective before it has been given a fair opportunity to act within its intended biological framework. On the other hand, misunderstanding melatonin as a conventional hypnotic may also encourage inappropriate dose escalation when immediate symptomatic improvement is not perceived. Such adjustments are unlikely to compensate for incorrect timing and may further increase pharmacokinetic variability, potentially obscuring the intended chronobiotic effect and complicating treatment evaluation [59,78].

## 6. Conclusions

The absence of a clear therapeutic framework creates a paradox in contemporary practice, where melatonin is both overused and underutilized: widely used for nonspecific insomnia with modest benefit yet underused or improperly applied in disorders where its chronobiotic properties are most relevant. This disconnection between rationale, clinical evidence and real-world practice defines the aforementioned “grey zones” of melatonin use. In our view, melatonin should be regarded as a medication rather than a benign dietary supplement and prescribed with the same diagnostic rigor applied to other pharmacological agents. Clinicians should first distinguish between insomnia and circadian rhythm disorders. When possible, they should use a regulated formulation and the lowest effective dose (often ≤1 mg for phase-shifting indications), avoid reflexive bedtime administration, and assess response only after two to three weeks of correctly timed and consistent use. Melatonin is most effective when integrated with behavioral and circadian interventions such as fixed wake times and appropriately timed light exposure, and when patients understand that its primary role is to shift biological timing rather than to induce immediate sedation. The field would also benefit from greater education of clinicians in circadian medicine, including an understanding of melatonin phase-response relationships and their implications for treatment timing. Such knowledge can then be effectively communicated to patients, whose understanding of the rationale for treatment is likely to improve adherence and clinical outcomes. Restoring this degree of precision may help align therapeutic expectations with melatonin’s true pharmacology and improve the rational use of one of the most widely used agents in sleep medicine.

## Data Availability

No new data were created or analyzed in this study. Data sharing is not applicable to this article.

## Abbreviations

The following abbreviations are used in this manuscript:

DSWPD	delayed sleep–wake phase disorder
REM	rapid eye movement
DLMO	dim-light melatonin onset
